# Clonal Seborrheic keratosis: A Diagnostic Dilemma

**DOI:** 10.5826/dpc.1104a95

**Published:** 2021-10-01

**Authors:** Satish Udare, Priyanka Patil

**Affiliations:** 1 Sparkle Skin & Aesthetic Clinic, Vashi, Navi Mumbai, India

**Keywords:** seborrheic keratosis, clonal, dermoscopy

## Introduction

Clonal seborrheic keratosis (SK) is an uncommon histological subtype of seborrheic keratosis which may resemble other benign or malignant lesions [1]. Herein, we highlight the clinical and histopathological features of this lesion and review the dermoscopic differential diagnoses.

## Case Presentation

**Figure 1:**
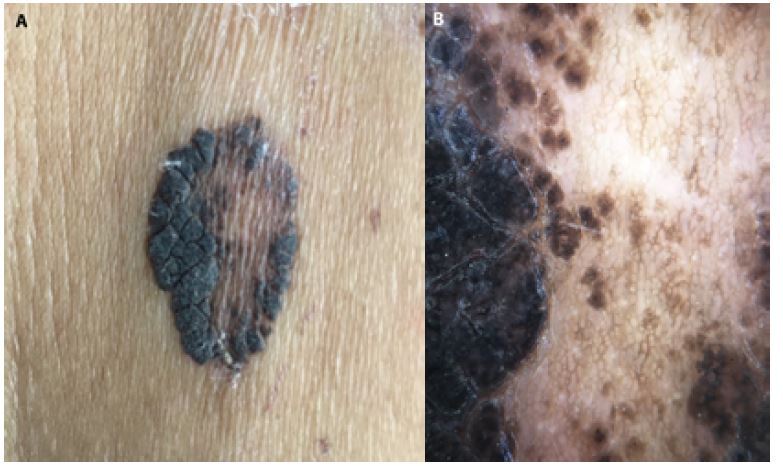
(A) Clinical presentation of clonal seborrheic keratosis. (B) Dermoscopy reveals the presence of globular-like structures and sharply demarcated borders.

**Figure 2:**
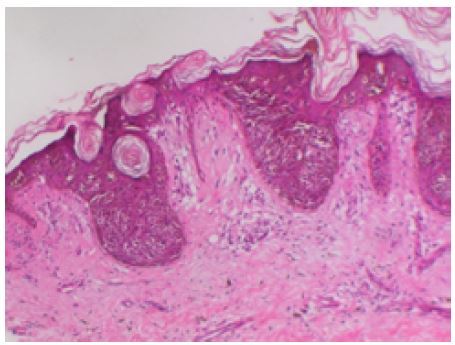
Well defined islands of basaloid cell nests within an acanthotic epidermis (H&E, X40).

A 65-year-old female presented with an asymptomatic dark colored lesion, that has been gradually increasing in size for the last 30 years on the left side of the lower back. No history of bleeding. No history of any medical illness or family history of skin malignancy. On examination single, well circumscribed, irregularly shaped, hyperpigmented keratotic plaque measuring 3 cm in maximum diameter (Figure 1). No palpable lymph nodes were detected. Dermoscopy showed the presence of bluish black to brown globules of varying size, irregularly distributed, well demarcated borders, and milia-like cysts (Figure 2). The tumor was biopsied. Histopathological examination showed epidermal hyperplasia with lamellated and basket weave orthohyperkeratosis with mild papillomatosis dermoscopically corresponding to fissures and ridges. Within the neoplasm two distinct subpopulations of basaloid cells (clones) were detected; one of them presented as a nest within the other (Figure 3). The nests dermoscopically corresponded to the globules. Occasional mitotic figures were seen within these clones. Abundant melanin was present within the nested subpopulation of keratinocytes. Sparse superficial perivascular lymphohistiocytic infiltrate is present. There was no interface change or lichenoid pattern to the infiltrate suggestive of clonal seborrheic keratosis. Immunostaining was not performed. Electrocautery of the lesion was performed with no relapse after 1 year of follow-up.

## Conclusion

In the dermoscopic differential diagnosis of clonal SK, we must consider Hidroacanthoma Simplex (HS) and its malignant variant, epidermal nevus, Pagetoid pigmented Bowen disease, in situ melanoma, and superficial Basal Cell Carcinoma (BCC). Clonal SK, is characterized by variously sized, blue-gray globular-like structures that are aggregated to form short lines or irregularly distributed within the lesion [2]. It can reveal other features suggestive of SK, including demarcated borders, milia-like cysts, comedo-like openings, and the jelly sign. Also, polymorphic vascular component is reported [2]. HS shows white globular structures surrounded by homogenous pigmented lines which are not seen in clonal SK.. Dermoscopy of a pigmented malignant hidracanthoma simplex arising from a HS reveals vessels in a conspicuous and irregular shape whereas in clonal SK glomerular, hairpin and dotted vessels are seen [3]. Epidermal nevus reveals large brown circles. In pagetoid pigmented Bowen disease, glomerular vessels, scaly surface, small brown globules regularly packed in a patchy distribution, and a grey homogenous pigmentation are seen. BCC has other characteristics such as arborizing vessels and maple leaf areas while coiled vessels are characteristic of melanoma.

The distinction between clonal SK and other benign or malignant lesions is challenging on dermoscopy. Histopathological examination will lead to accurate diagnosis in doubtful cases.

## References

[1] Bouhamed M, Bacha D, Abdelmoula F, Slama SB, Lahmar A, Bouraoui S, Sabeh MR (2019). Clonal seborrheic keratosis: a rare skin tumor. Pan Afr Med J.

[2] Longo C, Zalaudek I, Moscarella E, Lallas A, Piana S, Pellacani G (2014). Clonal seborrheic keratosis: dermoscopic and confocal microscopy characterization. J Eur Acad Dermatol Venereol.

[3] Ramyead S, Diaz-Cano SJ, Pozo-Garcia L (2015). Dermoscopy of clonal seborrheic keratosis. J Am Acad Dermatol.

